# Supplementary material for: PHIP suppresses NuRD to enable the growth of SWI/SNF-mutant cancers
Source: Nat Commun. 2026 Apr 7;17:2877. doi: 10.1038/s41467-026-70699-3 (PMC13057301; doi:10.1038/s41467-026-70699-3)
Supplement: Supplementary file 1 — Supplementary Information [file 41467_2026_70699_MOESM1_ESM.pdf]

Supplemental Table 1:

Reagents used for siRNA knockdown

Target	Supplier	Catalogue	Target Sequence
**PHIP B**	Dharmacon	J-019291-06-0010	UAAACUGACUGGCGGAUCA
**PHIP D**	Dharmacon	J-019291-08-0010	GAUGGGAGGUUGUUAGCUA
**siNT #1**	Dharmacon	D-001810-01-20	UGGUUUACAUGUCGACUAA
**siNT #2**	Dharmacon	D-001810-02-20	UGGUUUACAUGUUGUGUGA
**siCHD4 D**	Dharmacon	J-009774-08	GAAUAAAUUUCUAGCUCGA
**siRBBP4**	Dharmacon	J-012137-08-0020	GACUGAAUGUCUGGGAUUU

Supplemental Figure 1: Cancers with broad disruption of SWI/SNF are sensitive to PHIP inactivation. Related to Figure 1.

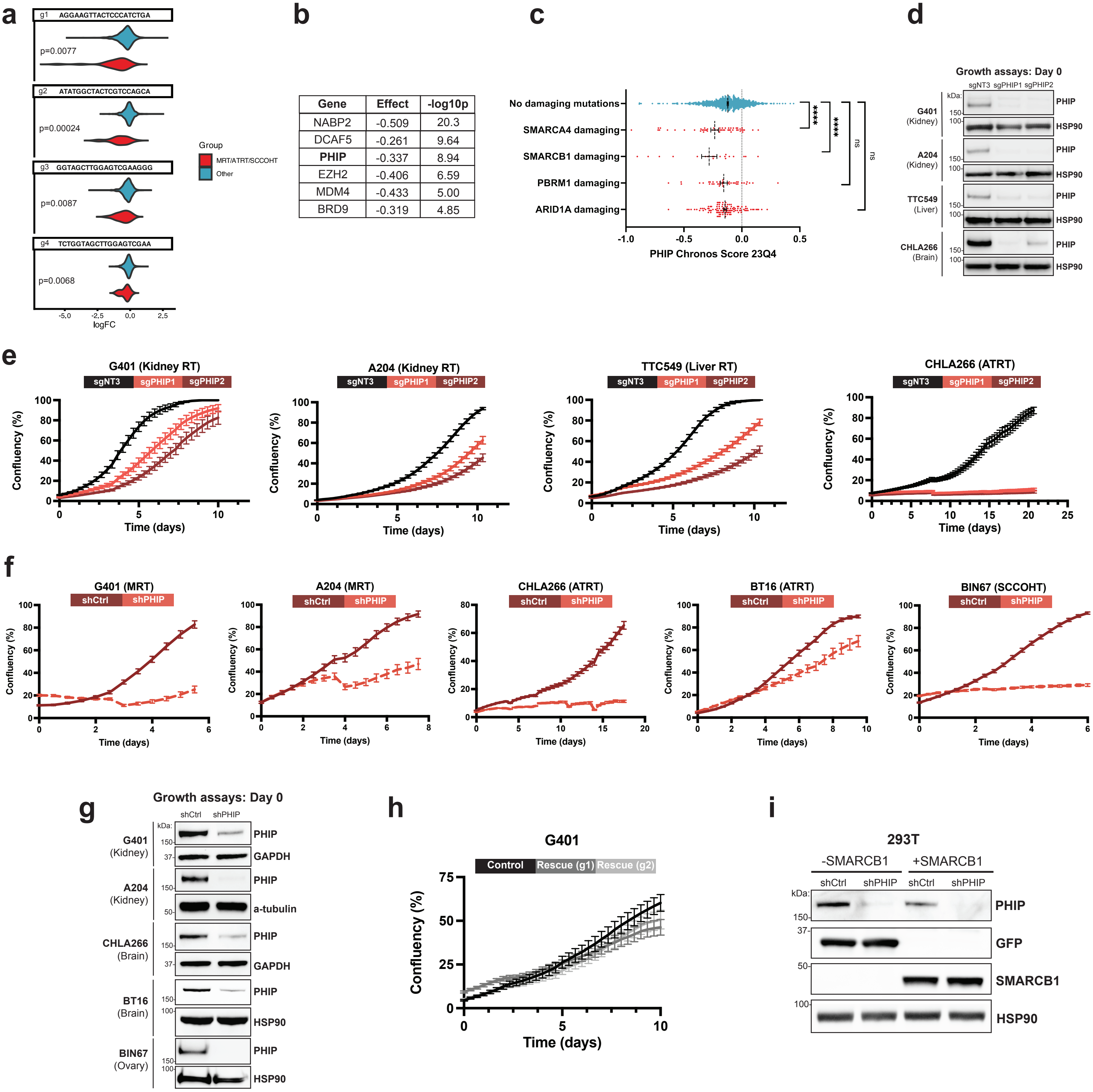

Gene	Effect	-log10p
NABP2	-0.509	20.3
DCAF5	-0.261	9.64
**PHIP**	-0.337	8.94
EZH2	-0.406	6.59
MDM4	-0.433	5.00
BRD9	-0.319	4.85

Supplementary Figure 1: Cancers with broad disruption of SWI/SNF are sensitive to PHIP inactivation

A: Violin plot comparing dependency scores for individual gRNAs ($n = 4$) targeting PHIP in RT and SCCOHT cell lines ($n = 19$) versus all other cell lines ($n = 1082$) in DepMap (24Q4 Chronos release). More negative scores indicate enhanced sensitivity to each sgRNA targeting PHIP. Statistical analysis was performed using a two-tailed Student's *t*-test. **B:** Effect size and significance score of several top hits from a two-class comparison of Chronos dependency scores of genes in RT cell lines ($n = 15$) compared to all other cell lines ($n = 1086$) in DepMap (24Q4 Chronos release). P-values were calculated using a two-tailed Benjamini–Hochberg-corrected Student's *t*-test. **C:** Scatter plot comparing dependency scores for PHIP in cancer cell lines with damaging mutations in SMARCA4 ($n = 52$), SMARCB1 ($n = 19$), PBRM1 ($n = 37$), or ARID1A ($n = 113$) versus all other cell lines ($n = 899$) in the DepMap (23Q4 Chronos release). Each point represents a cell line, and more negative scores indicate enhanced sensitivity to PHIP inactivation. Statistical analysis was performed using a two-tailed Student's *t*-test; ****$P < 0.0001$). The center line represents the mean, and the error bars represent the SEM. **D:** Western blot validation of PHIP knockout in cells used for the growth assays shown in panel E. Cell lysates were prepared at the start of the growth assays and immunoblotted for PHIP and the loading control HSP90. **E:** Plots of cell growth assays after transduction with two independent gRNAs targeting PHIP (red) or non-targeting gRNAs (black) in two kidney RT (malignant rhabdoid tumor, MRT) cell lines (G401 and A204), a liver RT cell line (TTC549), and a brain RT (atypical teratoid rhabdoid tumor, AT/RT) cell line (CHLA266). The plots depict confluency over time in days. Data are mean confluency measurements from $n = 8$ technical replicates, and error bars represent the SEM. **F:** Plots of cell growth assays after transduction with scramble shRNAs (dark red) or shRNAs targeting PHIP (light red) in MRT cell lines (G401 and A204), two AT/RT cell lines (CHLA266 and BT16), and one SCCOHT cell line (BIN67). The plots depict confluency over time in days. Data are mean confluency measurements from $n = 8$ technical replicates, and error bars represent the SEM. **G:** Western blot validation of PHIP knockdown in cells used for growth assays shown in panel F. Cell lysates were prepared at the start of growth assays and

immunoblotted for PHIP and the loading controls GAPDH, alpha-tubulin, or HSP90. **H:** Plot of cell growth assays in G401 RT cells transduced with two independent gRNAs targeting PHIP (gray) or non-targeting gRNAs (black) after expression of an sgRNA-resistant HA-PHIP construct. The plot depicts confluency over time in days. Data are mean confluency measurements from *n* = 8 technical replicates, and error bars represent the SEM. **I:** Western blots validating rescue of SMARCB1 and knockdown of PHIP in cells used for the 293T growth assay in Figure 1E. HSP90: loading control.

Supplemental Figure 2. PHIP activates transcription. Related to Figure 2.

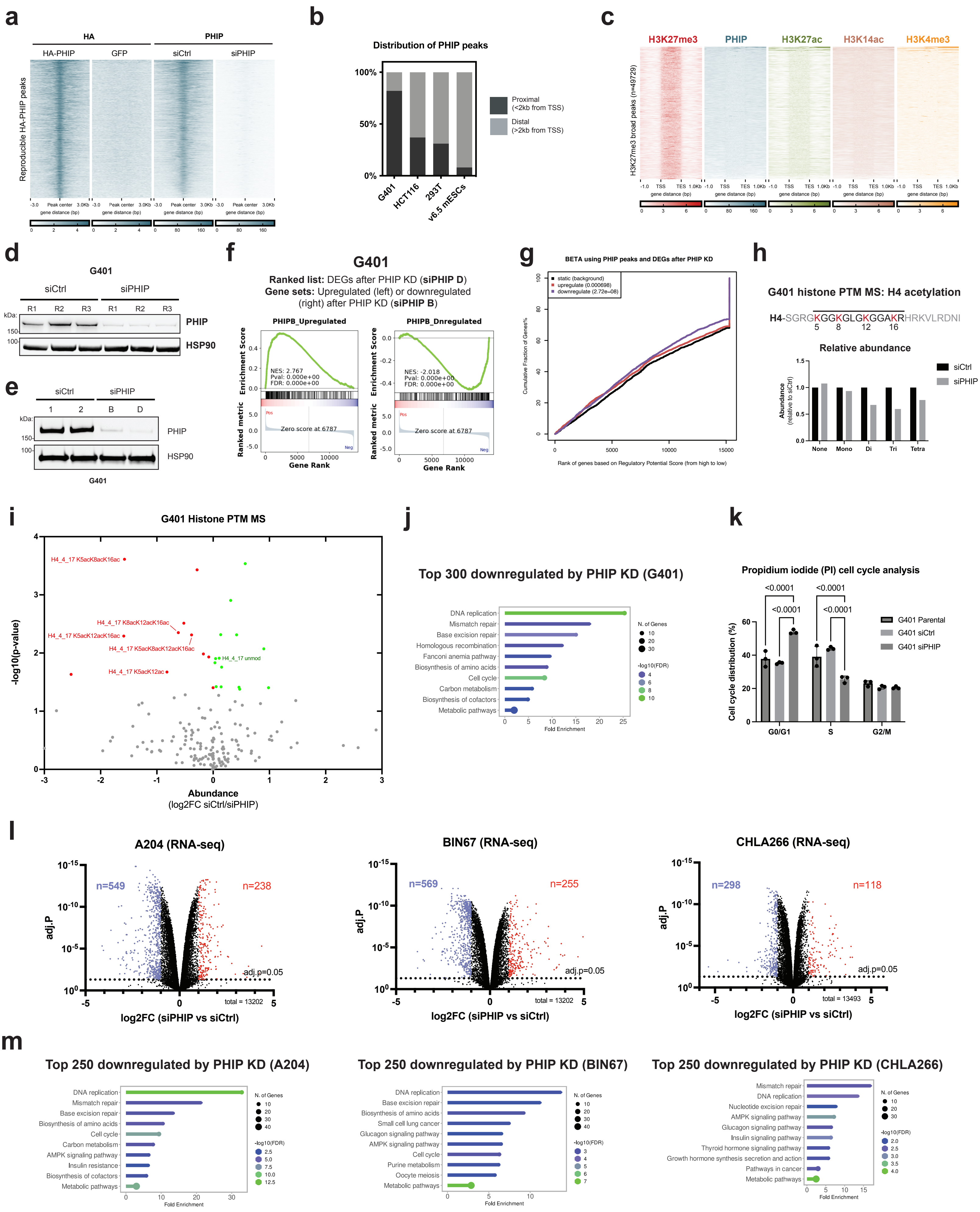

Supplementary Figure 2: PHIP activates transcription

A: Validation of HA-PHIP and PHIP ChIP-seq in RT cells. Heatmap of ChIP-seq signal at HA-PHIP peaks (*n* = 12243) for HA in G401 cells expressing HA-PHIP or GFP (empty vector control) (*n* = 2) and endogenous PHIP ChIP-seq in control and PHIP-knockdown G401 cells (*n* = 3). **B:** Bar chart illustrating PHIP peaks that are promoter-proximal (<2 kb from the TSS) versus distal (>2 kb from the TSS) in an RT cell line (G401) and in non-RT cell lines (HCT116 [GSM5696448], 293T [GSE101646], and v6.5 mESCs [GSE101646]). **C:** Heatmap of ChIP-seq signal for H3K27me3 (red, *n* = 3), PHIP (blue, *n* = 3), H3K27ac (green, *n* = 3), H3K14ac (salmon, *n* = 3), and H3K4me3 (orange, *n* = 3) at H3K27me3 peaks (*n* = 49,729) in G401 cells, sorted by PHIP intensity. **D:** Western blots confirming knockdown of PHIP in cells used for RNA-seq in G401 cells (*n* = 3 biological replicates). Loading control: HSP90. **E,F:** Validating PHIP knockdown and transcriptional changes after PHIP knockdown using two independent siRNAs. **(E)** Western blot confirming PHIP knockdown in G401 cells by using two independent siRNAs for RNA-seq. Loading control: HSP90. **(F)** Validating transcriptional changes observed after PHIP knockdown by using two independent siRNAs. Gene set enrichment analysis (GSEA) was performed using RNA-seq data after knockdown of PHIP in G401 cells by using two independent siRNAs. Ranked list (by logFC): differentially expressed genes after siPHIP D knockdown. Gene set: the top 250 genes upregulated (left) or downregulated (right) after siPHIP B knockdown. Strong enrichment of upregulated genes (NES: 2.767) and downregulated genes (NES: −2.018) was observed after knockdown of PHIP by using two independent siRNAs. **G:** Binding and Expression Target Analysis (BETA) comparing PHIP binding to changes in transcription in G401 cells. The red and purple lines represent activated and repressed genes, respectively, and the black line represents unchanged genes. Statistical analysis was performed using one-tailed Kolmogorov–Smirnov tests. Data are representative of *n* = 3 biological replicates. **H:** Bar chart summarizing mass spectrometry analysis of H4 peptide acetyl states (un-, mono-, di-, tri-, or tetra-acetyl) identified in control or PHIP-knockdown G401 cells (*n* = 3 biological replicates). Abundance is represented as relative to control. **I:** Volcano plot displaying histone posttranslational modification (PTM) changes measured by mass spectrometry of histones purified from

control and PHIP-knockdown G401 cells ($n = 3$ biological replicates). Statistical analysis was performed using a two-tailed Student's t-test. Each point represents a unique histone peptide. Peptides that become significantly less abundant are indicated in red (adjusted $P < 0.05$, log2FC < 0) and peptides that become significantly more abundant in green ($P < 0.05$, log2FC > 0). Peptides derived from H4 [amino acids 4−17] are labeled. **J:** Gene Ontology (GO) enrichment analysis of the top 300 downregulated genes (Log2FC<0, sorted by adjusted P value) after PHIP knockdown in G401 cells ($n = 3$ biological replicates). **K:** Bar graph of flow cytometry–based propidium iodide cell cycle analysis in parental, siCtrl-treated, and siPHIP-treated G401 RT cells (points represent $n = 3$ biological replicates). **L:** Volcano plot of differentially expressed genes after knocking down PHIP in A204 (kidney RT, left), BIN67 (SMARCA2/4-deficient SCCOHT, center), and CHLA266 (AT/RT, right) cells ($n = 3$ biological replicates). Statistical analysis was performed using a two-sided empirical Bayes moderated t-test using the limma package. The dashed line indicates adjusted $P = 0.05$. For significantly downregulated genes (blue): adjusted $P < 0.05$, log2FC < −1; for significantly upregulated genes (red): adjusted $P < 0.05$, log2FC > 1. **M:** GO enrichment analysis of the top 250 downregulated genes (Log2FC<0, sorted by adjusted P value) after PHIP knockdown in A204, BIN67, and CHLA266 cells ($n = 3$ biological replicates).

Supplemental Figure 3. PHIP recruits E3 ligases to chromatin and ubiquitinates NuRD. Related to Figure 3.

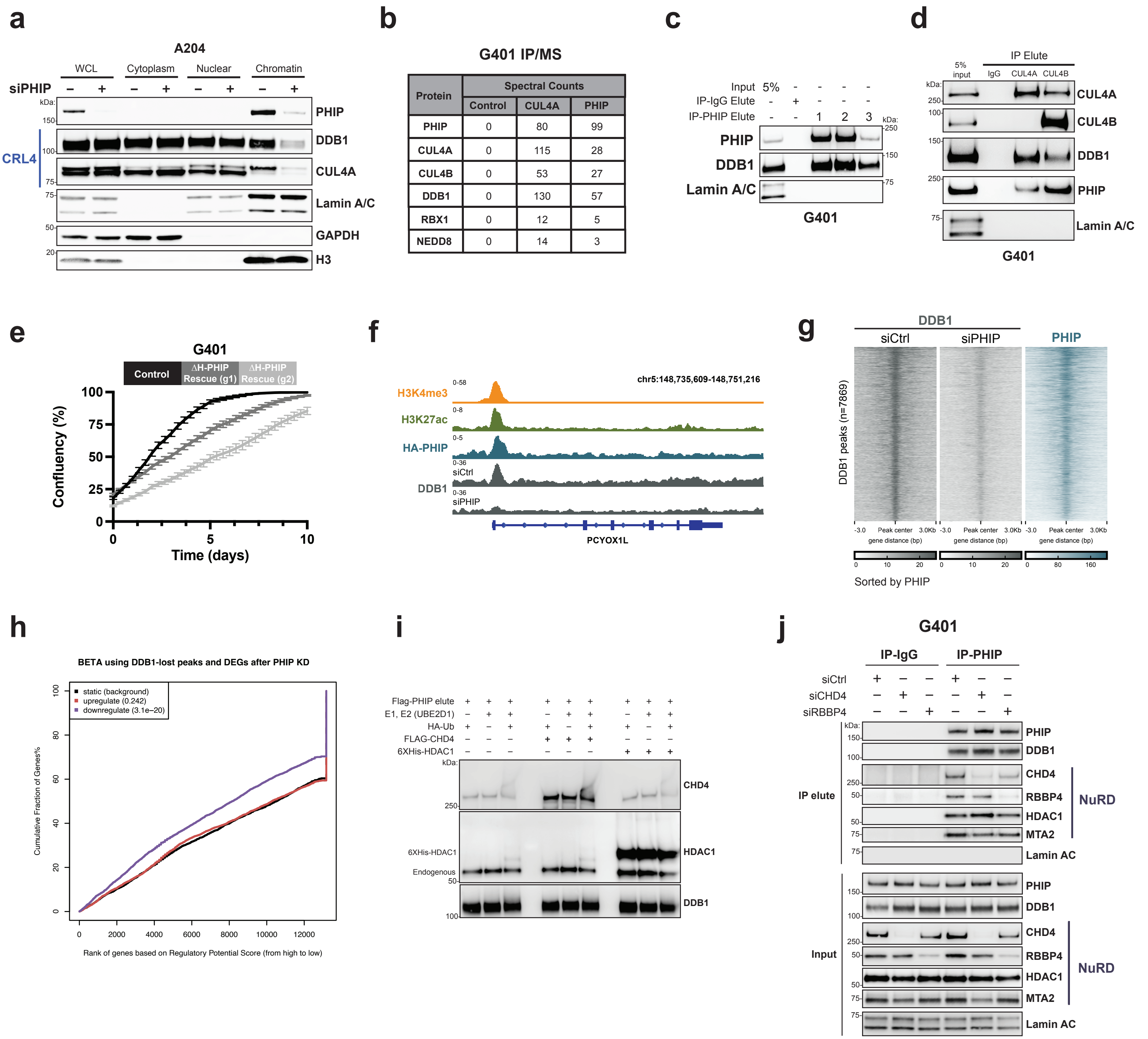

G401 IP/MS

Protein	Spectral Counts		
	Control	CUL4A	PHIP
PHIP	0	80	99
CUL4A	0	115	28
CUL4B	0	53	27
DDB1	0	130	57
RBX1	0	12	5
NEDD8	0	14	3

Supplementary Figure 3: PHIP recruits E3 ligases to chromatin and ubiquitinates NuRD

A: Western blots (WBs) of whole-cell lysate (WCL), and cytoplasmic, nuclear, and chromatin fractions with and without PHIP knockdown in A204 RT cells. GAPDH: cytoplasmic fraction loading control. Lamin A/C: nuclear soluble loading control. H3: chromatin loading control. Blots are representative of $n = 2$ biological replicates. **B:** Spectral counts for PHIP and members of the CRL4 complex from IP-MS after affinity purification of endogenous PHIP and CUL4A in G401 cells. **C,D:** Co-immunoprecipitations (co-IPs) and reverse co-IPs confirming the IP-MS results shown in panel B in G401 cells. Lamin A/C is a negative control. Data are representative of $n = 3$ biological replicates. **E:** Plot of cell growth assays in G401 RT cells transduced with two independent gRNAs targeting PHIP (gray) or with non-targeting gRNAs (black) after expression of an sgRNA-resistant HA-ΔH-PHIP construct. The plot depicts confluency over time in days. Data are mean confluency measurements from $n = 8$ technical replicates, and error bars represent the SEM. **F:** Example track showing the effect of PHIP knockdown on DDB1 (gray) at promoters as determined by ChIP-seq in G401 RT cells. Tracks for H3K4me3 (orange, $n = 3$), H3K27ac (green, $n = 3$), and HA-PHIP (blue, $n = 2$) are included. **G:** Heatmap of ChIP-seq signal for DDB1 (gray) in siCtrl-treated and siPHIP-treated G401 cells ($n = 3$) and for PHIP in G401 cells ($n = 3$) at all siCtrl DDB1 peaks ($n = 7869$). Sorted by PHIP signal strength. **H:** Binding and Expression Target Analysis (BETA) comparing the loss of DDB1 binding after PHIP knockdown to changes in transcription in G401 cells. The red and purple lines represent activated and repressed genes, respectively, and the black line represents unchanged genes. Statistical analysis was performed using one-tailed Kolmogorov–Smirnov tests. Data are representative of $n = 3$ biological replicates. **I:** Western blots of products from *in vitro* ubiquitination assays performed with FLAG-purified PHIP from 293T cells and recombinant CHD4 or HDAC1. Data are representative of $n = 3$ biological replicates. **J:** PHIP co-immunoprecipitation of NuRD subunits in siCtrl-treated, siCHD4-treated, and siRBBP4-treated G401 cells. Lamin A/C is a negative control, and the input shown is 2% of the lysate used for the co-IP study. Data are representative of $n = 3$ biological replicates.

Supplemental Figure 4. PHIP suppresses silencing by NuRD complexes at promoters. Related to Figure 4.

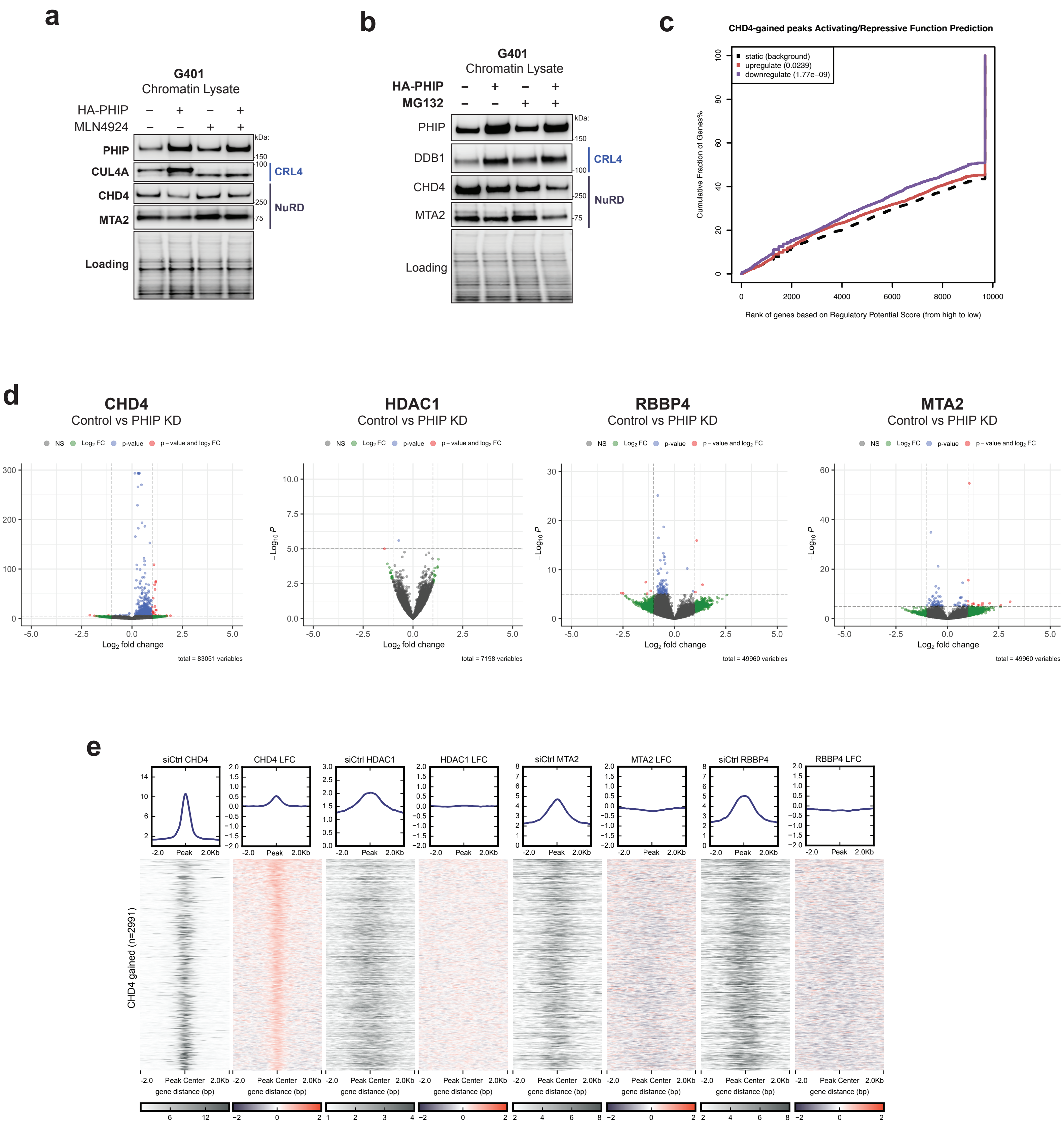

Supplementary Figure 4: PHIP suppresses silencing by NuRD complexes at promoters

A,B: Western blots of chromatin lysate after overexpression of full-length PHIP and treatment with (A) neddylation inhibitor (MLN-4924) or (B) proteosome inhibitor (MG132) in G401 RT cells. Total protein stain is included as a loading control. Blot is representative of n = 3 biological replicates. **C:** Binding and Expression Target Analysis (BETA) comparing the gain of CHD4 binding after PHIP knockdown to changes in transcription in G401 cells. The red and purple lines represent activated and repressed genes, respectively, and the black line represents unchanged genes. Statistical analysis was performed using one-tailed Kolmogorov–Smirnov tests. Data are representative of $n = 3$ biological replicates. **D:** Volcano plots displaying differential binding changes measured by ChIP-seq for the indicated targets (CHD4, HDAC1, RBBP4, and MTA2) in control and PHIP-knockdown G401 cells ($n = 3$ biological replicates). Each point represents a reproducible peak for the indicated target. Statistical analysis was performed using a moderated t-test in the limma package. The horizontal dashed line indicates adjusted $P = 0.05$, and the vertical dashed lines indicate log2FC = −1 (left) and log2FC = 1 (right). Green points represent peaks with log2FC > 1 or log2FC < −1 but $P > 0.05$. Blue points represent peaks with $P < 0.05$ but log2FC values between −1 and 1. Red points represent peaks with log2FC > 1 or log2FC < −1 and $P < 0.05$. **E:** ChIP-seq for additional NuRD subunits (RBBP4, MTA2, and HDAC1) after PHIP knockdown in G401 RT cells. Heatmap showing ChIP-seq coverage in control conditions (gray, $n = 3$) and log2FC after PHIP knockdown (navy/red) for CHD4, HDAC1, MTA2, and RBBP4 at CHD4-gained peaks ($n = 2991$). Sorted by CHD4 LFC.

Supplemental Figure 5. Patient-derived, in vivo models of RT specifically depend upon PHIP. Related to Figure 6.

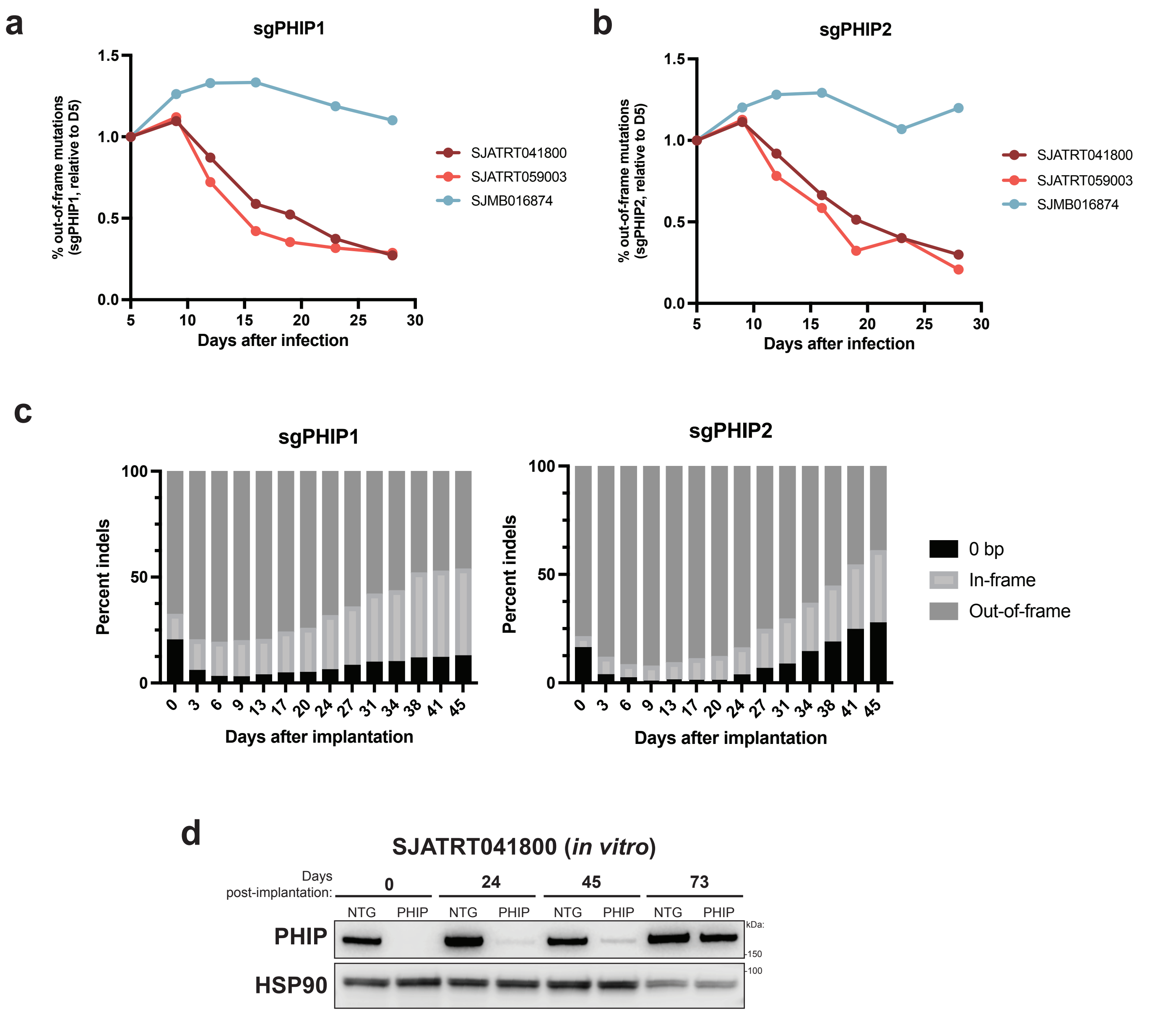

Supplementary Figure 5: Patient-derived *in vivo* models of RT specifically depend upon PHIP

A, B: Deep sequencing of tumor organoids from the flow-based fitness assay depicted in Figure 6B. Plot of relative abundance of damaging, out-of-frame mutations at the sgPHIP1 **(A)** or sgPHIP2 **(B)** locus over time in two AT/RT tumor organoid lines (SJATRT041800 and SJATRT059003, red) compared to a medulloblastoma tumor organoid line with intact SWI/SNF (SJMB016874, blue). **C:** Deep sequencing of tumor organoids grown in culture *in vitro* after orthotopic xenograft implantation into mice. Bar chart showing the ratio of 0-bp (black), in-frame (light gray), and out-of-frame (dark gray) indels at the sgPHIP1 and sgPHIP2 target loci over time. **D:** Western blots of tumor organoids grown in culture *in vitro* after implantation. HSP90: loading control.
