# Supplementary material for: PHIP suppresses NuRD to enable the growth of SWI/SNF-mutant cancers
Source: Nat Commun. 2026 Apr 7;17:2877. doi: 10.1038/s41467-026-70699-3 (PMC13057301; doi:10.1038/s41467-026-70699-3)
Supplement: Supplementary file 2 — Description of Additional Supplementary Files [file 41467_2026_70699_MOESM2_ESM.pdf]

Description of Additional Supplementary Files

Supplementary Data 1. Details on antibodies used in this study
