# Supplementary material for: PHIP suppresses NuRD to enable the growth of SWI/SNF-mutant cancers
Source: Nat Commun. 2026 Apr 7;17:2877. doi: 10.1038/s41467-026-70699-3 (PMC13057301; doi:10.1038/s41467-026-70699-3)
Supplement: Supplementary file 4 — Reporting Summary [file 41467_2026_70699_MOESM4_ESM.pdf]

nature portfolio

Corresponding author(s): Charles W. M. Roberts

Last updated by author(s): Feb 12, 2026

Reporting Summary

Nature Portfolio wishes to improve the reproducibility of the work that we publish. This form provides structure for consistency and transparency in reporting. For further information on Nature Portfolio policies, see our Editorial Policies and the Editorial Policy Checklist.

Statistics

For all statistical analyses, confirm that the following items are present in the figure legend, table legend, main text, or Methods section.

n/a	Confirmed	
☐	☒	The exact sample size (*n*) for each experimental group/condition, given as a discrete number and unit of measurement
☒	☐	A statement on whether measurements were taken from distinct samples or whether the same sample was measured repeatedly
☐	☒	The statistical test(s) used AND whether they are one- or two-sided<br>*Only common tests should be described solely by name; describe more complex techniques in the Methods section.*
☐	☒	A description of all covariates tested
☐	☒	A description of any assumptions or corrections, such as tests of normality and adjustment for multiple comparisons
☐	☒	A full description of the statistical parameters including central tendency (e.g. means) or other basic estimates (e.g. regression coefficient) AND variation (e.g. standard deviation) or associated estimates of uncertainty (e.g. confidence intervals)
☐	☒	For null hypothesis testing, the test statistic (e.g. *F*, *t*, *r*) with confidence intervals, effect sizes, degrees of freedom and *P* value noted<br>*Give P values as exact values whenever suitable.*
☒	☐	For Bayesian analysis, information on the choice of priors and Markov chain Monte Carlo settings
☒	☐	For hierarchical and complex designs, identification of the appropriate level for tests and full reporting of outcomes
☒	☐	Estimates of effect sizes (e.g. Cohen's *d*, Pearson's *r*), indicating how they were calculated

Our web collection on statistics for biologists contains articles on many of the points above.

Software and code

Policy information about availability of computer code

Data collection	Cell viability/proliferation: Incucyte Live-Cell Analysis System (Essen BioScience).<br>Western blots imaging: BioRad ChemiDoc or Licor Odyssey XF and analyzed with ImageLab or ImageStudio software, respectively<br>Trim-Galore (v0.4.4,) cutadapt, FastQC, bwa aln bwa samse (v0.7.12-r103963), samtools, STAR (2.7.1a65), biobambam2 (v2.0.8766). SPP (v1.1167) bedtools (v2.24.068) UCSC tools (v469) MACS2, pybedtools (v0.8.168,72), edgeR, limma-voom, R, deeptools, HOMER (v4.9.1), RSEM, BETA, GSEApy (v1.1.2). We performed an input scaled spike in normalization using custom code that will be deposited on github when the paper is published.
Data analysis	Sequence data was processed as reported previously(Radko-Juettner et al. 2024). Adapters were trimmed from raw reads in FASTQ files with Trim-Galore (v0.4.4, https://www.bioinformatics.babraham.ac.uk/projects/trim_galore/), using the cutadapt program(Martin 2011). QC was performed on trimmed files by using FastQC (https://www.bioinformatics.babraham.ac.uk/projects/fastqc/) with the quality score cutoff set to Q20. Paired-end ChIP-seq reads were mapped to a hybrid human–Drosophila reference genome (merged hg19/GRCh37.p13 and dm6 genomes) with bwa aln, followed by bwa samse (v0.7.12-r1039(Li and Durbin 2009)) with the -K flag set to 10,000,000. The output was converted to BAM format with samtools (v1.2(Li et al. 2009)), and reads mapped to the human reference genome were extracted for further analysis by using samtools. RNA-seq paired-end reads were mapped to the human reference genome (hg19/GRCh37.p13) with STAR (2.7.1a(Dobin et al. 2013)). Duplicated reads were identified and marked using the bamsormadup tool from biobambam2 (v2.0.87(Tischler and Leonard 2014)).<br>Cross-correlation analysis was conducted with SPP (v1.11(Kharchenko, Tolstorukov, and Park 2008)), and uniquely mapped reads were extracted with samtools(Li et al. 2009), extended with BEDtools (v2.24.0(Quinlan and Hall 2010)), using the fragment size calculated by the cross-correlation analysis. UCSC tools (v4(Kuhn, Haussler, and Kent 2013)) was used to convert bam files to bigWig track files. Peaks were called using MACS2 (v2.2.7.1(Zhang et al. 2008)) with -nomodel -q 0.05 flags (high confidence peaks). Low-confidence narrow peaks were also

called using a more relaxed criterion (-q 0.5 flag). Reproducible peaks of biological replicates were called using a previously reported approachCross-correlation analysis was conducted with SPP (v1.11(Kharchenko, Tolstorukov, and Park 2008)), and uniquely mapped reads were extracted using samtools(Li et al. 2009), extended with BEDtools (v2.24.0(Quinlan and Hall 2010)), using the fragment size calculated by the cross-correlation analysis. UCSC tools (v4(Kuhn, Haussler, and Kent 2013)) was used to convert bam files to bigWig track files. Peaks were called using MACS(Zhang et al. 2008) with -nomodel -q 0.05 flags (high confidence peaks). Low-confidence narrow peaks were also called using a more relaxed criterion (-q 0.5 flag). Reproducible peaks of biological replicates were called using a previously reported approach(Wang et al. 2022). Reproducible peaks were defined as those loci with overlapping high-confidence peaks in more than one replicate or as loci called as high-confidence peaks in one replicate and as high- or low-confidence peaks in all other replicates. The coordinates of the final reproducible peaks were based on the union of coordinates of the overlapping high-confidence peaks. For visualization on heatmaps, peaks that overlapped with annotated TSSs were identified using intersectBed.
Drosophila reads were extracted from hybrid bam files by using samtools (v1.2(Li et al. 2009)) and the dm6 chromosome names (v1.2). The number of uniquely aligned, non-duplicated dm6 reads was used to compute the % spike in reads over all reads sequenced for each sample and input. A ratio of dm6 reads was computed for each sample, compared to the dm6 reads generated for each input sample. Scaling factors were computed as the input scaled dm6 ratio for each sample, divided by the largest dm6 ratio for the mark. For generating bigWig files, a library size normalization was included, defined as 15 million / uniquely aligned, non-duplicate reads * the previously computed scaling factor. Code for spike-input normalization has been uploaded to GitHub: [https://github.com/jamyers2358/Malone_PHIP_SWISNF_Dependency].
Combined count matrix files of reads within each reference peak were quantified using the intersect command from pybedtools (v0.8.1(Quinlan and Hall 2010; Dale, Pedersen, and Quinlan 2011)) and were used as input for downstream differential testing in R (v4.3.3/ v4.5.0). To perform statistical tests of differences among experimental groups, the trimmed mean of M-value scale factors was estimated using edgeR (v0.16) and a limma-voom (v3.58.1) empirical Bayes moderation to establish significant differences. Significant differential binding of targets was defined as significantly gained (log2FC>0 and FDR<0.05) or significantly lost (log2FC<0 and FDR<0.05). The scaling factors calculated above were applied during differential testing for input-spike-in–scaled experiments.
For visualization of ChIP-seq samples without spike normalization, the density of mapped reads was converted to bigWig format and normalized to 15 million non-duplicated mapped reads to normalize for library size. Input-spike–normalized samples were library-size normalized, defined as 15 million / uniquely aligned, non-duplicate reads, and multiplied by the previously computed scaling factor. The signal for the three biological replicates was averaged for display in the main figure panels. Differential bigWigs were generated with averaged bigWig files, using the log2FC function of bigwigCompare. All heatmaps and metaplots of normalized, averaged coverage were generated using deepTools (v2.5.3(Ramírez et al. 2014)), using reference point mode. Matrix files were generated with computeMatrix, heatmaps with plotHeatmap, and metaplots with plotProfile. The integrative genome viewer (IGV v 2.11.3(Thorvaldsdóttir, Robinson, and Mesirov 2013)) was used to visualize coverage at specific loci.
Reproducible peaks were annotated using HOMER (v4.9.1). Peaks assigned to genes and within ±2 kb of their promoters were defined as "proximal peaks." Peaks more than 2 kb from annotated TSSs were defined as "distal peaks." Several publicly available PHIP ChIP-seq datasets were used (HCT116(Morgan et al. 2021) [GSM5696448], 293T(Morgan et al. 2017) [GSE101646], and v6.5 mESCs(Morgan et al. 2017) [GSE101646]).
Gene-level counts were quantified using rsem-calculate-expression, using BAM files generated by STAR. A combined count matrix with all samples was generated and used as input for differential testing. To perform statistical tests of differences between experimental groups, the trimmed mean of M-value scale factors was estimated using edgeR and a limma-voom empirical Bayes moderation to establish significant differences. Significantly differentially expressed genes (DEGs) were defined as those with log2FC>1 and FDR<0.05 or log2FC<1 and FDR< 0.05. Hierarchically clustered and z-score–centered heatmaps were generated using pheatmap (https://cran.r-project.org/web/packages/pheatmap/index.html). Gene Ontology enrichment analysis was performed with the indicated gene sets by using ShinyGO (v0.82, http://bioinformatics.sdstate.edu/go/).
BETA (v1.0.7(Wang et al. 2013)) was run using ChIP-seq binding peaks and significantly differentially expressed genes. The -d flag was set to 100,000 to include only peaks within 100 kb of a gene's TSS. The regulatory potential score was calculated using Sg=∑ki=1e−(0.5 +4Δi)Sg=∑ik=1e−(0.5+4Δi). All peaks (k) within 100 kb of the TSS were considered. The distance between a binding site and the TSS is Δ, which is proportional to 100 kb. P values were calculated by the Kolmogorov–Smirnov test to measure the significance of the upregulated genes group or the downregulated genes group relative to the static genes group.
Pre-ranked gene set enrichment analysis (GSEA) was performed in python by using GSEApy(Fang, Liu, and Peltz 2022) (v1.1.2, https://gseapy.readthedocs.io/en/latest/introduction.html). Gene log2FC was used to rank genes in the gene list. Several custom gene sets were defined using internal and external differential expression analyses.

For manuscripts utilizing custom algorithms or software that are central to the research but not yet described in published literature, software must be made available to editors and reviewers. We strongly encourage code deposition in a community repository (e.g. GitHub). See the Nature Portfolio guidelines for submitting code & software for further information.

Data

Policy information about availability of data

All manuscripts must include a data availability statement. This statement should provide the following information, where applicable:

- Accession codes, unique identifiers, or web links for publicly available datasets
- A description of any restrictions on data availability
- For clinical datasets or third party data, please ensure that the statement adheres to our policy

The RNA and ChIP-Seq data generated in this study have been deposited in the Gene Expression Omnibus (GEO) database under accession GSE315219 [https://www.ncbi.nlm.nih.gov/geo/query/acc.cgi?acc=GSE315219] and GSE299672 [https://www.ncbi.nlm.nih.gov/geo/query/acc.cgi?acc=GSE299672]. Previously published ChIP-seq and RNA-seq datasets used in this study can be accessed under the following GEO accession codes: GSE101646 [https://www.ncbi.nlm.nih.gov/geo/query/acc.cgi?acc=GSE101646], GSE189235 [https://www.ncbi.nlm.nih.gov/geo/query/acc.cgi?acc=GSE189235], GSE180487 [https://www.ncbi.nlm.nih.gov/geo/query/acc.cgi?acc=GSE180487], GSE210636 [https://www.ncbi.nlm.nih.gov/geo/query/acc.cgi?acc=GSE210636], GSE178490 [https://www.ncbi.nlm.nih.gov/geo/query/acc.cgi?acc=GSE178490], and GSE215024 [https://www.ncbi.nlm.nih.gov/geo/query/acc.cgi?acc=GSE215024]. Results from the CRISPR screen can be accessed at [https://depmap.org/portal/]. The raw IP-MS and histone-MS data generated in this study have been deposited to ProteomeXchange under accession code PXD069047 [https://massive.ucsd.edu/ProteoSAFe/dataset.jsp?accession=MSV000099353]. Plasmids obtained from Addgene are available under the indicated accession numbers, and plasmids generated in this study are available from the corresponding author upon reasonable request. All other raw data supporting the findings of this study are included in the Source Data file.

Research involving human participants, their data, or biological material

Policy information about studies with human participants or human data. See also policy information about sex, gender (identity/presentation), and sexual orientation and race, ethnicity and racism.

Reporting on sex and gender	*Use the terms sex (biological attribute) and gender (shaped by social and cultural circumstances) carefully in order to avoid confusing both terms. Indicate if findings apply to only one sex or gender; describe whether sex and gender were considered in study design; whether sex and/or gender was determined based on self-reporting or assigned and methods used. Provide in the source data disaggregated sex and gender data, where this information has been collected, and if consent has been obtained for sharing of individual-level data; provide overall numbers in this Reporting Summary. Please state if this information has not been collected. Report sex- and gender-based analyses where performed, justify reasons for lack of sex- and gender-based analysis.*
Reporting on race, ethnicity, or other socially relevant groupings	*Please specify the socially constructed or socially relevant categorization variable(s) used in your manuscript and explain why they were used. Please note that such variables should not be used as proxies for other socially constructed/relevant variables (for example, race or ethnicity should not be used as a proxy for socioeconomic status). Provide clear definitions of the relevant terms used, how they were provided (by the participants/respondents, the researchers, or third parties), and the method(s) used to classify people into the different categories (e.g. self-report, census or administrative data, social media data, etc.) Please provide details about how you controlled for confounding variables in your analyses.*
Population characteristics	*Describe the covariate-relevant population characteristics of the human research participants (e.g. age, genotypic information, past and current diagnosis and treatment categories). If you filled out the behavioural & social sciences study design questions and have nothing to add here, write "See above."*
Recruitment	*Describe how participants were recruited. Outline any potential self-selection bias or other biases that may be present and how these are likely to impact results.*
Ethics oversight	*Identify the organization(s) that approved the study protocol.*

Note that full information on the approval of the study protocol must also be provided in the manuscript.

Field-specific reporting

Please select the one below that is the best fit for your research. If you are not sure, read the appropriate sections before making your selection.

☒ Life sciences ☐ Behavioural & social sciences ☐ Ecological, evolutionary & environmental sciences

For a reference copy of the document with all sections, see nature.com/documents/nr-reporting-summary-flat.pdf

Life sciences study design

All studies must disclose on these points even when the disclosure is negative.

Sample size	For all NGS experiments, growth proliferation assays and immunoblots, at least 3 biological replicates were performed. This sample size is sufficient for capturing biological variability and powering us to identify significant differences between binding and gene expression. Three patient-derived tumor organoid models were used for fitness assays in vitro, and 1 model was orthotopically xenografted and studied in vivo.
Data exclusions	No data were excluded from this study.
Replication	For all experiments we routinely apply analyses that evaluate sample relatedness including correlation, PCA, and/or MDS to understand replicate concordance. We do not state any findings that were not replicated.
Randomization	Cells were always chosen randomly
Blinding	N.A

Reporting for specific materials, systems and methods

We require information from authors about some types of materials, experimental systems and methods used in many studies. Here, indicate whether each material, system or method listed is relevant to your study. If you are not sure if a list item applies to your research, read the appropriate section before selecting a response.

Materials & experimental systems

n/a	Involved in the study	
☐	☒	Antibodies
☐	☒	Eukaryotic cell lines
☒	☐	Palaeontology and archaeology
☐	☒	Animals and other organisms
☒	☐	Clinical data
☒	☐	Dual use research of concern
☒	☐	Plants

Methods

n/a	Involved in the study	
☐	☒	ChIP-seq
☐	☒	Flow cytometry
☒	☐	MRI-based neuroimaging

Antibodies

Antibodies used

Anti-PHIP (Rabbit Polyclonal, Bethyl, A302-055A, Lot 1) (1:1000 WB)
https://www.thermofisher.com/antibody/product/PHIP-Antibody-Polyclonal/A302-055A

Anti-PHIP (Rabbit Polyclonal, Novus, NBP2-33883, Lot 28457) (1:1000 WB, 1:50 IP)
https://www.novusbio.com/products/phip-antibody_nbp2-33883?
srsltid=AfmBOooFc5gtRhiJTjsO6Tom947BjBLg9HTqkgHqVoKIsmTPW_JkDdUj

Anti-GFP (Rabbit Polyclonal, Abcam, ab290, Lot 1068770-2) (1:1000 WB)
https://www.abcam.com/en-us/products/primary-antibodies/gfp-antibody-ab290

Anti-SMARCB1 (Rabbit Monoclonal, Cell Signaling Technology, 91735, Lot 1) (1:3000 WB)
https://www.cellsignal.com/products/primary-antibodies/smarcb1-baf47-d8m1x-rabbit-monoclonal-antibody/91735?
srsltid=AfmBOoo6N9f0rjFqUWJAV93irj89FrHJ0Ao73LnoPrCN_6bYzq1b_Bk_

Anti-HSP90 (Rabbit Monoclonal, Cell Signaling Technology, 4877, Lot 7) (1:2000 WB)
https://www.cellsignal.com/products/primary-antibodies/hsp90-c45g5-rabbit-monoclonal-antibody/4877?srsltid=AfmBOord302sJj-SCSTh57yj1TJsS-uY2cdFTmOpp-6veJSCKjJnKdFl

Anti-Alpha Tubulin (Rabbit Polyclonal, Cell Signaling Technology, 2144, Lot 6) (1:1000 WB)
https://www.cellsignal.com/products/primary-antibodies/alpha-tubulin-antibody/2144?
srsltid=AfmBOoqpLybNcmQHO83J8dP8ethTcAwXj7wPpXewm2KYe3SDK3BxfFyB

Anti-GAPDH (Mouse Monoclonal, Cell Signaling Technology, 97166, Lot 6) (1:3000 WB)
https://www.cellsignal.com/products/primary-antibodies/gapdh-d4c6r-mouse-monoclonal-antibody/97166?
srsltid=AfmBOopJXRc3__6U93y3jR7tzQQ-tTCzBfy1kJHH5xV7YGxuSpMi3VQP

Anti-Beta Actin (Mouse Monoclonal, Sigma, A5441, Lot 079M4799V) (1: 3000 WB)
https://www.sigmaaldrich.com/US/en/product/sigma/a5441?
srsltid=AfmBOoo55tQjD4X2-4VPPfsuVANN-2iBrXQhbac4cCsHKLb1HRTHMwhf

Anti-Lamin A/C (Mouse Monoclonal, Cell Signaling Technology, 4777, Lot 7) (1:1000 WB)
https://www.cellsignal.com/products/primary-antibodies/lamin-a-c-4c11-mouse-monoclonal-antibody/4777?
srsltid=AfmBOoqXblv3IoPY56AOYfcG3WVBuZqrmxy5uHB9W3U_K0OAAFUoCCV-

Anti-DDB1 (Rabbit Monoclonal, Abcam, ab109027, Lot 1018022-6) (1:1000 WB, 1:50 ChIP)
https://www.citeab.com/antibodies/761059-ab109027-anti-ddb1-antibody-epr6089

Anti-CUL4A (Rabbit Polyclonal, Cell Signaling Technology, 2699, Lot 4) (1:1000 WB, 1:50 IP)
https://www.cellsignal.com/products/primary-antibodies/cul4a-antibody/2699?
srsltid=AfmBOorpCJUNkG4AyyF55cz9eIEUTWWF3RQMXYZCXyJ4dTe6tLexXs6q

Anti-CUL4B (Rabbit Polyclonal, Proteintech, 12916-1-AP, Lot 116837) (1:1000 WB, 1:100 IP)
https://www.ptglab.com/products/CUL4B-Antibody-12916-1-AP.htm?
srsltid=AfmBOopYhGxIaqTEUaNLG4xAbJpuECGoxpZ9Wokdjt5SOqRmiTRTENf9

Anti-H4 (Mouse Monoclonal, Cell Signaling Technology, 2935, Lot 6) (1:2000 WB)
https://www.cellsignal.com/products/primary-antibodies/histone-h4-l64c1-mouse-monoclonal-antibody/2935?
srsltid=AfmBOoqO3wKTB2oij4B5rRzoQieWtZxZdDqdowiKU7O_UXb7L4CkMKUs

Anti-H3 (Mouse Monoclonal, Cell Signaling Technology, 14269, Lot 8) (1:10000 for immunoblots)
https://www.cellsignal.com/products/primary-antibodies/histone-h3-1b1b2-mouse-monoclonal-antibody/14269?
srsltid=AfmBOoprqQUn6E2IXuiUAFPO-_FIQcp-AFeeqcyATsdlsDDQXFBfqDv6

Anti-H3K14ac (Rabbit Monoclonal, Abcam, ab52946, Lot 1029948-24, 1001631-4) (1:2000 WB, 7:1000 ChIP)
https://www.abcam.com/en-us/products/primary-antibodies/histone-h3-acetyl-k14-antibody-ep964y-chip-grade-ab52946

Anti-H3K27ac (Rabbit Polyclonal, Abcam, ab4729, Lot 1058174-1) (1: 2000 WB, 5:1000 ChIP)
https://www.abcam.com/en-us/products/primary-antibodies/histone-h3-acetyl-k27-antibody-chip-grade-ab4729

Anti-H4K12ac (Rabbit Polyclonal, Active Motif, 39066, Lot 16119002) (1:2000 WB)
https://www.activemotif.com/catalog/details/39165.html

Anti-CHD4 (Rabbit Monoclonal, Cell Signaling Technology, 12011) (1:1000 WB)
https://www.cellsignal.com/products/primary-antibodies/chd4-d4b7-rabbit-monoclonal-antibody/12011?srsltid=AfmBOopcnkwRH3LOBmQEv0liEnH1yvIOpao-jGftR3eN2Xv139QJnjTO

Anti-CHD4 (Rabbit Monoclonal, Abcam, ab240640, Lot 1043705-7, 11211685-1) (1:1000 WB, 1:100 IP, 8:1000 ChIP)
https://www.abcam.com/en-us/products/primary-antibodies/chd4-antibody-epr22953-38-chip-grade-ab240640

Anti-RBBP4 (Rabbit Monoclonal, Abcam, ab1765, Lot 108913-1) (1:1000 WB)
https://www.abcam.com/en-us/products/primary-antibodies/rbbp4-antibody-ab1765

Anti-RBBP4 (Rabbit Monoclonal, Abcam, ab79416, Lot GR3418888-4) (1:1000 WB, 1:100 IP, 1:100 ChIP)
https://www.abcam.com/en-us/products/primary-antibodies/rbbp4-antibody-epr3411-chip-grade-ab79416

Anti-HDAC1 (Mouse Polyclonal, Cell Signaling Technology, 5356, Lot 7) (1:1000 WB)
https://www.cellsignal.com/products/primary-antibodies/hdac1-10e2-mouse-monoclonal-antibody/5356?srsltid=AfmBOoqCvo5NvNH18YkltcNJai-sXOfvCoTlsu9w1T_-MJZ1SalChsLB

Anti-HDAC1 (Rabbit Polyclonal, Active Motif, 40967, Lot 24046163-1) (1:1000 WB, 5:1000 ChIP)
https://www.activemotif.com/catalog/details/40967/hdac1-antibody-pab

Anti-HDAC2 (Rabbit Monoclonal, Cell Signaling Technology, 57156, Lot 1) (1:1000 WB)
https://www.cellsignal.com/products/primary-antibodies/hdac2-d6s5p-rabbit-monoclonal-antibody/57156?srsltid=AfmBOorQtUDXrHfq5rg0It6JyeRe0666d81SyPJk-SFoFlv-beOQBhS3

Anti-MBD3 (Rabbit Monoclonal, Abcam, ab157464, Lot 1016754-20) (1:1000 WB)
https://www.abcam.com/en-us/products/primary-antibodies/mbd3-antibody-epr9913-chip-grade-ab157464

Anti-MTA2 (Rabbit Polyclonal, Abcam, ab8106, Lot 1058517-8) (1:1000 WB, 5:1000 ChIP)
https://www.abcam.com/en-us/products/primary-antibodies/mta2-pid-antibody-ab8106

Anti-GATAD2A (Rabbit Monoclonal, Cell Signaling Technology, 17705, Lot 1) (1:1000 WB)
https://www.cellsignal.com/products/primary-antibodies/gatad2a-e7b5b-rabbit-monoclonal-antibody/17705?srsltid=AfmBOopz2-m4z1VallJQDZt74NQL0BwpcQi-rtu4YXmWTk3NLjfbnGqY

Anti-SMARCC1 (Rabbit Monoclonal, Cell Signaling Technology, 11956, Lot 5) (1:1000 WB)
https://www.cellsignal.com/products/primary-antibodies/smarcc1-baf155-d7f8s-rabbit-monoclonal-antibody/11956?srsltid=AfmBOoqIGzQ373wHVrnOon5GdPvvz9iohoIM5dA7bOGgqmasGEI6YBBS

Anti-SMARCA4 (Rabbit Monoclonal, Cell Signaling Technology, 49360, Lot 3) (1:1000 WB)
https://www.cellsignal.com/products/primary-antibodies/brg1-d1q7f-rabbit-monoclonal-antibody/49360?srsltid=AfmBOorH9gc2vYtpHFcSstpB5l7soPiGgonkWHlZ8yeslviC-x5GnLn3

Anti-SMARCA2 (Rabbit Monoclonal, Cell Signaling Technology, 11966, Lot 6) (1:1000 WB)
https://www.cellsignal.com/products/primary-antibodies/brm-d9e8b-rabbit-monoclonal-antibody/11966?srsltid=AfmBOoq4VbYUsoQfl-h12iFmaq4s-SiqUiND77i7PKvt1NfVifVS4odX

Anti-IgG (Rabbit Polyclonal, Cell Signaling Technology, 2729, Lot 11) (1:200 IP)
https://www.cellsignal.com/products/primary-antibodies/normal-rabbit-igg/2729?srsltid=AfmBOoqALCNMrOGj52aQkMqUElXqDyx-IVOs_Dgm0_6ATUmVUv48RI9K

Anti-HA (Rabbit Monoclonal, Cell Signaling Technology, 3724, Lot 11) (15:1000 ChIP)
https://www.cellsignal.com/products/primary-antibodies/ha-tag-c29f4-rabbit-monoclonal-antibody/3724?srsltid=AfmBOooo_OoULVwUHsqUpyAY2p4NLMc40GMBTn7IFCn5KRL96WMn8NSa

Anti-H3K4me3 (Rabbit Polyclonal, Abcam, ab8580, Lot GR3425198-1) (5:1000 ChIP)
https://www.abcam.com/en-us/products/primary-antibodies/histone-h3-tri-methyl-k4-antibody-chip-grade-ab8580

Anti-H3K27me3 (Rabbit Monoclonal, Cell Signaling Technology, 9733, Lot 19) (1:50 ChIP)
https://www.cellsignal.com/products/primary-antibodies/tri-methyl-histone-h3-lys27-c36b11-rabbit-monoclonal-antibody/9733?srsltid=AfmBOoobqW7shMUPIyN19B5YN17DETdFaijyCPtoUb6VKvV6Q_CR7iHy

Anti-H4ac (Rabbit Polyclonal, Millipore, 06-866, Lot 4049811) (15:1000 ChIP)
https://www.merckmillipore.com/HK/en/product/Anti-acetyl-Histone-H4-Antibody,MM_NF-06-866

Anti-PHIP (Rabbit, Ali Shilatifard, Northwestern University) (1:50 ChIP)
https://pmc.ncbi.nlm.nih.gov/articles/PMC8653789/

AffiniPure Goat Anti-Rabbit IgG (H+L) (Rabbit Polyclonal, Jackson Immuno Research, 111-035-003, Lot 173071) (1:10000 WB)
https://www.jacksonimmuno.com/catalog/products/111-035-003

AffiniPure Goat Anti-Mouse IgG (H+L) (Jackson Immuno Research, 115-035-003, Lot 172297) (1:10000 WB)
https://www.jacksonimmuno.com/catalog/products/115-035-003

Validation	We used all antibodies under manufacturers' recommended conditions and/or based on multiple publications. Links for validation included above.

Eukaryotic cell lines

Policy information about cell lines and Sex and Gender in Research

Cell line source(s)	G401 (ATCC-CRL1441), A204 (ATCC-HTB-82), and HEK293T (ATCC-CRL-3216) cell lines were obtained from ATCC. Kelly cells were provided by Adam Durbin at St. Jude Children's Research Hospital. BIN67 were provided by Bernard E. Weissman at the University of North Carolina. CHLA266 was provided by the Children's Oncology Group. BT16 cells were obtained through a material transfer agreement from C. D. James. SJATRT041800 and SJATRT059003 tumoroids were obtained from the lab of Dr. Martine Roussel at St. Jude Children's Research Hospital. TTC549 cells were provided by Timothy Triche at UCLA.
Authentication	Commerical cell lines from ATCC were authenticated by manufacturer and we authenticated by STR profiling G401, A204, BIN67, TTC549, Kelly, and CHLA-266.
Mycoplasma contamination	Our lab routinely tests for mycoplasma contamination using Gelantis MycoScope PCR Mycoplasma Detection Kit (Cat#MY01100). Cell lines used in this study are mycoplasma free
Commonly misidentified lines (See ICLAC register)	N.A

Animals and other research organisms

Policy information about studies involving animals; ARRIVE guidelines recommended for reporting animal research, and Sex and Gender in Research

Laboratory animals	8-12 weeks naïve female CD1-nude mice (Charles River #086NU/NUCD1)
Wild animals	N.A
Reporting on sex	All animals used were female. The utilization of male mice in randomization can often result in aggressive behavior that can interfere with the studies.
Field-collected samples	N.A
Ethics oversight	All animal studies were approved by the St. Jude Children's Research Hospital Animal Care and Use Committee and performed in accordance with best practices outlined by the NIH Office of Laboratory Animal Welfare.

Note that full information on the approval of the study protocol must also be provided in the manuscript.

Plants

Seed stocks	*Report on the source of all seed stocks or other plant material used. If applicable, state the seed stock centre and catalogue number. If plant specimens were collected from the field, describe the collection location, date and sampling procedures.*
Novel plant genotypes	*Describe the methods by which all novel plant genotypes were produced. This includes those generated by transgenic approaches, gene editing, chemical/radiation-based mutagenesis and hybridization. For transgenic lines, describe the transformation method, the number of independent lines analyzed and the generation upon which experiments were performed. For gene-edited lines, describe the editor used, the endogenous sequence targeted for editing, the targeting guide RNA sequence (if applicable) and how the editor was applied.*
Authentication	*Describe any authentication procedures for each seed stock used or novel genotype generated. Describe any experiments used to assess the effect of a mutation and, where applicable, how potential secondary effects (e.g. second site T-DNA insertions, mosiacism, off-target gene editing) were examined.*

ChIP-seq

Data deposition

☒ Confirm that both raw and final processed data have been deposited in a public database such as GEO.

☒ Confirm that you have deposited or provided access to graph files (e.g. BED files) for the called peaks.

Data access links *May remain private before publication.*	https://www.ncbi.nlm.nih.gov/geo/query/acc.cgi?acc=GSE315219 - parent<br><br>https://www.ncbi.nlm.nih.gov/geo/query/acc.cgi?acc=GSE299672 - ChIP-seq

https://www.ncbi.nlm.nih.gov/geo/query/acc.cgi?acc=GSE299562 - RNA-seq

Files in database submission

GSE299672 A novel NuRD regulator is essential in SWI/SNF-mutant cancers [ChIP-seq] Jun 11, 2029 approved None
GSM9043371 G401_input_siCtrl_1_histone Jun 11, 2029 approved BW
GSM9043372 G401_input_siPHIP_1_histone Jun 11, 2029 approved BW
GSM9043373 G401_H3K4me3ab8580_siCtrl_1 Jun 11, 2029 approved BW NARROWPEAK
GSM9043374 G401_H3K4me3ab8580_siCtrl_2 Jun 11, 2029 approved BW NARROWPEAK
GSM9043375 G401_H3K4me3ab8580_siCtrl_3 Jun 11, 2029 approved BW NARROWPEAK
GSM9043376 G401_H3K4me3ab8580_siPHIP_1 Jun 11, 2029 approved BW NARROWPEAK
GSM9043377 G401_H3K4me3ab8580_siPHIP_2 Jun 11, 2029 approved BW NARROWPEAK
GSM9043378 G401_H3K4me3ab8580_siPHIP_3 Jun 11, 2029 approved BW NARROWPEAK
GSM9043379 G401_H3K27me3cst9733S_siCtrl_1 Jun 11, 2029 approved BW BED
GSM9043380 G401_H3K27me3cst9733S_siCtrl_2 Jun 11, 2029 approved BW BED
GSM9043381 G401_H3K27me3cst9733S_siCtrl_3 Jun 11, 2029 approved BW BED
GSM9043382 G401_H3K27me3cst9733S_siPHIP_1 Jun 11, 2029 approved BW BED
GSM9043383 G401_H3K27me3cst9733S_siPHIP_2 Jun 11, 2029 approved BW BED
GSM9043384 G401_H3K27me3cst9733S_siPHIP_3 Jun 11, 2029 approved BW BED
GSM9043385 G401_HAcst3724S_GFP_1 Jun 11, 2029 approved BW
GSM9043386 G401_HAcst3724S_GFP_2 Jun 11, 2029 approved BW
GSM9043387 G401_HAcst3724S_HA-PHIP_1 Jun 11, 2029 approved BW NARROWPEAK
GSM9043388 G401_HAcst3724S_HA-PHIP_2 Jun 11, 2029 approved BW NARROWPEAK
GSM9043389 G401_DDB1ab109027_siCtrl_1 Jun 11, 2029 approved NARROWPEAK BW
GSM9043390 G401_DDB1ab109027_siCtrl_2 Jun 11, 2029 approved NARROWPEAK BW
GSM9043391 G401_DDB1ab109027_siCtrl_3 Jun 11, 2029 approved NARROWPEAK BW
GSM9043392 G401_DDB1ab109027_siPHIP_1 Jun 11, 2029 approved NARROWPEAK BW
GSM9043393 G401_DDB1ab109027_siPHIP_2 Jun 11, 2029 approved NARROWPEAK BW
GSM9043394 G401_DDB1ab109027_siPHIP_3 Jun 11, 2029 approved NARROWPEAK BW
GSM9043395 G401_input_siCtrl_1_DDB1 Jun 11, 2029 approved BW
GSM9043396 G401_input_siPHIP_1_DDB1 Jun 11, 2029 approved BW
GSM9043397 G401_H3K14ac-ab52946_siCtrl_1 Jun 11, 2029 approved BW NARROWPEAK
GSM9043398 G401_H3K14ac-ab52946_siCtrl_2 Jun 11, 2029 approved BW NARROWPEAK
GSM9043399 G401_H3K14ac-ab52946_siCtrl_3 Jun 11, 2029 approved BW NARROWPEAK
GSM9043400 G401_H3K14ac-ab52946_siPHIP_1 Jun 11, 2029 approved BW NARROWPEAK
GSM9043401 G401_H3K14ac-ab52946_siPHIP_2 Jun 11, 2029 approved BW NARROWPEAK
GSM9043402 G401_H3K14ac-ab52946_siPHIP_3 Jun 11, 2029 approved BW NARROWPEAK
GSM9043403 G401_input_siCtrl_1_H3K14ac Jun 11, 2029 approved BW
GSM9043404 G401_input_siPHIP_1_H3K14ac Jun 11, 2029 approved BW
GSM9043405 G401_H4ac06866_siCtrl_1 Jun 11, 2029 approved NARROWPEAK BW
GSM9043406 G401_H4ac06866_siCtrl_2 Jun 11, 2029 approved NARROWPEAK BW
GSM9043407 G401_H4ac06866_siCtrl_3 Jun 11, 2029 approved NARROWPEAK BW
GSM9043408 G401_H4ac06866_siPHIP_1 Jun 11, 2029 approved NARROWPEAK BW
GSM9043409 G401_H4ac06866_siPHIP_2 Jun 11, 2029 approved NARROWPEAK BW
GSM9043410 G401_H4ac06866_siPHIP_3 Jun 11, 2029 approved NARROWPEAK BW
GSM9043411 G401_inputH4ac_siCtrl_1 Jun 11, 2029 approved BW
GSM9043412 G401_inputH4ac_siPHIP_1 Jun 11, 2029 approved BW
GSM9043413 G401_H3K27acab4729_siCtrl_1_inputscale Jun 11, 2029 approved NARROWPEAK BW
GSM9043414 G401_H3K27acab4729_siCtrl_2_inputscale Jun 11, 2029 approved NARROWPEAK BW
GSM9043415 G401_H3K27acab4729_siCtrl_3_inputscale Jun 11, 2029 approved NARROWPEAK BW
GSM9043416 G401_H3K27acab4729_siPHIP_1_inputscale Jun 11, 2029 approved NARROWPEAK BW
GSM9043417 G401_H3K27acab4729_siPHIP_2_inputscale Jun 11, 2029 approved NARROWPEAK BW
GSM9043418 G401_H3K27acab4729_siPHIP_3_inputscale Jun 11, 2029 approved NARROWPEAK BW
GSM9043419 G401_CHD4-ab240640_siCtrl_1 Jun 11, 2029 approved NARROWPEAK BW
GSM9043420 G401_CHD4-ab240640_siCtrl_2 Jun 11, 2029 approved NARROWPEAK BW
GSM9043421 G401_CHD4-ab240640_siCtrl_3 Jun 11, 2029 approved NARROWPEAK BW
GSM9043422 G401_CHD4-ab240640_siPHIP_1 Jun 11, 2029 approved NARROWPEAK BW
GSM9043423 G401_CHD4-ab240640_siPHIP_2 Jun 11, 2029 approved NARROWPEAK BW
GSM9043424 G401_CHD4-ab240640_siPHIP_3 Jun 11, 2029 approved NARROWPEAK BW
GSM9043425 G401_inputH3K27_siCtrl_1 Jun 11, 2029 approved BW
GSM9043426 G401_inputH3K27_siPHIP_1 Jun 11, 2029 approved BW
GSM9043427 G401_inputCHD4_siCtrl_1 Jun 11, 2029 approved BW
GSM9043428 G401_inputCHD4_siPHIP_1 Jun 11, 2029 approved BW
GSM9043429 G401_inputHDAC1_siCtrl_1 Jun 11, 2029 approved BW
GSM9043430 G401_inputHDAC1_siCtrl_2 Jun 11, 2029 approved BW
GSM9043431 G401_inputHDAC1_siCtrl_3 Jun 11, 2029 approved BW
GSM9043432 G401_inputHDAC1_siPHIP_1 Jun 11, 2029 approved BW
GSM9043433 G401_inputHDAC1_siPHIP_2 Jun 11, 2029 approved BW
GSM9043434 G401_inputHDAC1_siPHIP_3 Jun 11, 2029 approved BW
GSM9043435 G401_HDAC1-40967_siCtrl_1 Jun 11, 2029 approved NARROWPEAK BW
GSM9043436 G401_HDAC1-40967_siCtrl_2 Jun 11, 2029 approved NARROWPEAK BW
GSM9043437 G401_HDAC1-40967_siCtrl_3 Jun 11, 2029 approved NARROWPEAK BW
GSM9043438 G401_HDAC1-40967_siPHIP_1 Jun 11, 2029 approved NARROWPEAK BW
GSM9043439 G401_HDAC1-40967_siPHIP_2 Jun 11, 2029 approved NARROWPEAK BW
GSM9043440 G401_HDAC1-40967_siPHIP_3 Jun 11, 2029 approved NARROWPEAK BW

GSM9043441 G401_RBBP4ab79416_siCtrl_1 Jun 11, 2029 approved NARROWPEAK BW
GSM9043442 G401_RBBP4ab79416_siCtrl_2 Jun 11, 2029 approved NARROWPEAK BW
GSM9043443 G401_RBBP4ab79416_siCtrl_3 Jun 11, 2029 approved NARROWPEAK BW
GSM9043444 G401_RBBP4ab79416_siPHIP_1 Jun 11, 2029 approved NARROWPEAK BW
GSM9043445 G401_RBBP4ab79416_siPHIP_2 Jun 11, 2029 approved NARROWPEAK BW
GSM9043446 G401_RBBP4ab79416_siPHIP_3 Jun 11, 2029 approved NARROWPEAK BW
GSM9043447 G401_MTA2ab8106_siCtrl_1 Jun 11, 2029 approved NARROWPEAK BW
GSM9043448 G401_MTA2ab8106_siCtrl_2 Jun 11, 2029 approved NARROWPEAK BW
GSM9043449 G401_MTA2ab8106_siCtrl_3 Jun 11, 2029 approved NARROWPEAK BW
GSM9043450 G401_MTA2ab8106_siPHIP_1 Jun 11, 2029 approved NARROWPEAK BW
GSM9043451 G401_MTA2ab8106_siPHIP_2 Jun 11, 2029 approved NARROWPEAK BW
GSM9043452 G401_MTA2ab8106_siPHIP_3 Jun 11, 2029 approved NARROWPEAK BW
GSM9043453 G401_input_siCtrl_1_MTA2_RBBP4 Jun 11, 2029 approved BW
GSM9043454 G401_input_siCtrl_2_MTA2_RBBP4 Jun 11, 2029 approved BW
GSM9043455 G401_input_siCtrl_3_MTA2_RBBP4 Jun 11, 2029 approved BW
GSM9043456 G401_input_siPHIP_1_MTA2_RBBP4 Jun 11, 2029 approved BW
GSM9043457 G401_input_siPHIP_2_MTA2_RBBP4 Jun 11, 2029 approved BW
GSM9043458 G401_input_siPHIP_3_MTA2_RBBP4 Jun 11, 2029 approved BW
GSM9438255 3404887_G401_H3K27ac-ab4729_control_1_ChIP Jun 11, 2029 approved BW NARROWPEAK
GSM9438256 3404888_G401_H3K27ac-ab4729_control_2_ChIP Jun 11, 2029 approved BW NARROWPEAK
GSM9438257 3404889_G401_H3K27ac-ab4729_control_3_ChIP Jun 11, 2029 approved BW NARROWPEAK
GSM9438258 3404890_G401_H3K27ac-ab4729_PHIP-OE_1_ChIP Jun 11, 2029 approved BW NARROWPEAK
GSM9438259 3404891_G401_H3K27ac-ab4729_PHIP-OE_2_ChIP Jun 11, 2029 approved BW NARROWPEAK
GSM9438260 3404892_G401_H3K27ac-ab4729_PHIP-OE_3_ChIP Jun 11, 2029 approved BW NARROWPEAK
GSM9438261 3404893_G401_H3K27ac-ab4729_PHIP-KD_1_ChIP Jun 11, 2029 approved BW NARROWPEAK
GSM9438262 3404894_G401_H3K27ac-ab4729_PHIP-KD_2_ChIP Jun 11, 2029 approved BW NARROWPEAK
GSM9438263 3404895_G401_H3K27ac-ab4729_PHIP-KD_3_ChIP Jun 11, 2029 approved BW NARROWPEAK
GSM9438264 3404896_G401_H3K27ac-ab4729_PHIP-rescue_1_ChIP Jun 11, 2029 approved BW NARROWPEAK
GSM9438265 3404897_G401_H3K27ac-ab4729_PHIP-rescue_2_ChIP Jun 11, 2029 approved BW NARROWPEAK
GSM9438266 3404898_G401_H3K27ac-ab4729_PHIP-rescue_3_ChIP Jun 11, 2029 approved BW NARROWPEAK
GSM9438267 3404915_G401_input_control_1_ChIP Jun 11, 2029 approved BW
GSM9438268 3404916_G401_input_control_2_ChIP Jun 11, 2029 approved BW
GSM9438269 3404917_G401_input_control_3_ChIP Jun 11, 2029 approved BW
GSM9438270 3404918_G401_input_PHIP-OE_1_ChIP Jun 11, 2029 approved BW
GSM9438271 3404919_G401_input_PHIP-OE_2_ChIP Jun 11, 2029 approved BW
GSM9438272 3404920_G401_input_PHIP-OE_3_ChIP Jun 11, 2029 approved BW
GSM9438273 3404921_G401_input_PHIP-KD_1_ChIP Jun 11, 2029 approved BW
GSM9438274 3404922_G401_input_PHIP-KD_2_ChIP Jun 11, 2029 approved BW
GSM9438275 3404923_G401_input_PHIP-KD_3_ChIP Jun 11, 2029 approved BW
GSM9438276 3404924_G401_input_PHIP-rescue_1_ChIP Jun 11, 2029 approved BW
GSM9438277 3404925_G401_input_PHIP-rescue_2_ChIP Jun 11, 2029 approved BW
GSM9438278 3404926_G401_input_PHIP-rescue_3_ChIP Jun 11, 2029 approved BW
GSM9442374 3128867_G401_PHIPmarc_siCtrl_1_ChIP Jun 11, 2029 approved NARROWPEAK BW
GSM9442375 3128868_G401_PHIPmarc_siCtrl_2_ChIP Jun 11, 2029 approved NARROWPEAK BW
GSM9442376 3128869_G401_PHIPmarc_siCtrl_3_ChIP Jun 11, 2029 approved NARROWPEAK BW
GSM9442377 3128873_G401_PHIPmarc_siPHIP_1_ChIP Jun 11, 2029 approved NARROWPEAK BW
GSM9442378 3128874_G401_PHIPmarc_siPHIP_2_ChIP Jun 11, 2029 approved NARROWPEAK BW
GSM9442379 3128875_G401_PHIPmarc_siPHIP_3_ChIP Jun 11, 2029 approved NARROWPEAK BW
GSM9442380 3128876_G401_PHIPinput_siCtrl_1_ChIP Jun 11, 2029 approved BW
GSM9442381 3128877_G401_PHIPinput_siPHIP_1_ChIP Jun 11, 2029 approved BW
GSM9442382 3334381_G401_CHD4ab240640_GFP_1_ChIP Jun 11, 2029 approved BW NARROWPEAK
GSM9442383 3334382_G401_CHD4ab240640_GFP_2_ChIP Jun 11, 2029 approved BW NARROWPEAK
GSM9442384 3334383_G401_CHD4ab240640_GFP_3_ChIP Jun 11, 2029 approved BW NARROWPEAK
GSM9442385 3334384_G401_CHD4ab240640_SMARCB1_1_ChIP Jun 11, 2029 approved BW NARROWPEAK
GSM9442386 3334385_G401_CHD4ab240640_SMARCB1_2_ChIP Jun 11, 2029 approved BW NARROWPEAK
GSM9442387 3334386_G401_CHD4ab240640_SMARCB1_3_ChIP Jun 11, 2029 approved BW NARROWPEAK
GSM9442388 3334387_G401_input_GFP_1_ChIP Jun 11, 2029 approved BW
GSM9442389 3334388_G401_input_GFP_2_ChIP Jun 11, 2029 approved BW
GSM9442390 3334389_G401_input_GFP_3_ChIP Jun 11, 2029 approved BW
GSM9442391 3334390_G401_input_SMARCB1_1_ChIP Jun 11, 2029 approved BW
GSM9442392 3334391_G401_input_SMARCB1_2_ChIP Jun 11, 2029 approved BW
GSM9442393 3334392_G401_input_SMARCB1_3_ChIP Jun 11, 2029 approved BW

Genome browser session
(e.g. UCSC)

N.A

Methodology

Replicates

We always design experiments to be n = 3 when statistical comparisons are made

Sequencing depth

197106141 207745746 2697821_G401_DDB1ab109027_siCtrl_1_ChIP_HAM
216014423 236820076 2697822_G401_DDB1ab109027_siCtrl_2_ChIP_HAM
218189693 241798328 2697823_G401_DDB1ab109027_siCtrl_3_ChIP_HAM
233171801 259803636 2697824_G401_DDB1ab109027_siPHIP_1_ChIP_HAM

238436911 274171926 2697825_G401_DDB1ab109027_siPHIP_2_ChIP_HAM
222378680 247406120 2697826_G401_DDB1ab109027_siPHIP_3_ChIP_HAM
234597807 240792746 2697827_G401_input_siCtrl_1_ChIP_HAM
247948919 255636406 2697828_G401_input_siPHIP_1_ChIP_HAM
174502631 178074592 3220431_G401_H3K27acab4729_siCtrl_1_ChIP_HAM
143239703 146209710 3220432_G401_H3K27acab4729_siCtrl_2_ChIP_HAM
139280453 141865764 3220433_G401_H3K27acab4729_siCtrl_3_ChIP_HAM
161665532 163854570 3220434_G401_H3K27acab4729_siPHIP_1_ChIP_HAM
169237431 172174808 3220435_G401_H3K27acab4729_siPHIP_2_ChIP_HAM
183533191 187271454 3220436_G401_H3K27acab4729_siPHIP_3_ChIP_HAM
125475815 136704530 3220437_G401_CHD4-ab240640_siCtrl_1_ChIP_HAM
140654176 153434756 3220438_G401_CHD4-ab240640_siCtrl_2_ChIP_HAM
137994320 147777288 3220439_G401_CHD4-ab240640_siCtrl_3_ChIP_HAM
133555298 143970038 3220440_G401_CHD4-ab240640_siPHIP_1_ChIP_HAM
160116469 170599090 3220441_G401_CHD4-ab240640_siPHIP_2_ChIP_HAM
145447706 157482934 3220442_G401_CHD4-ab240640_siPHIP_3_ChIP_HAM
163985759 167428618 3220443_G401_inputH3K27_siCtrl_1_ChIP_HAM
173173548 176874136 3220444_G401_inputH3K27_siPHIP_1_ChIP_HAM
164457327 168154398 3220445_G401_inputCHD4_siCtrl_1_ChIP_HAM
125688269 128442660 3220446_G401_inputCHD4_siPHIP_1_ChIP_HAM
230252018 259672292 3198078_G401_H4ac06866_siCtrl_1_ChIP_HAM
202224485 219715708 3198079_G401_H4ac06866_siCtrl_2_ChIP_HAM
222144478 246599110 3198080_G401_H4ac06866_siCtrl_3_ChIP_HAM
169391933 183433010 3198081_G401_H4ac06866_siPHIP_1_ChIP_HAM
199111743 219509482 3198082_G401_H4ac06866_siPHIP_2_ChIP_HAM
259420952 291879034 3198083_G401_H4ac06866_siPHIP_3_ChIP_HAM
165213043 170196986 3198084_G401_inputH4ac_siCtrl_1_ChIP_HAM
204025412 210368578 3198085_G401_inputH4ac_siPHIP_1_ChIP_HAM
167526149 172675096 3198086_G401_inputHDAC1_siCtrl_1_ChIP_HAM
186948240 192961764 3198087_G401_inputHDAC1_siCtrl_2_ChIP_HAM
208634483 215806494 3198088_G401_inputHDAC1_siCtrl_3_ChIP_HAM
216817808 224284692 3198089_G401_inputHDAC1_siPHIP_1_ChIP_HAM
210008125 217189828 3198090_G401_inputHDAC1_siPHIP_2_ChIP_HAM
175322229 184869250 3198091_G401_inputHDAC1_siPHIP_3_ChIP_HAM
213813584 234800894 3198092_G401_HDAC1-40967_siCtrl_1_ChIP_HAM
211883129 241048110 3198093_G401_HDAC1-40967_siCtrl_2_ChIP_HAM
199238760 226705654 3198094_G401_HDAC1-40967_siCtrl_3_ChIP_HAM
191560721 216673074 3198095_G401_HDAC1-40967_siPHIP_1_ChIP_HAM
214041663 240714462 3198096_G401_HDAC1-40967_siPHIP_2_ChIP_HAM
227971759 264187990 3198097_G401_HDAC1-40967_siPHIP_3_ChIP_HAM
182091513 197005586 3274161_G401_RBBP4ab79416_siCtrl_1_ChIP_HAM
183054573 195301726 3274162_G401_RBBP4ab79416_siCtrl_2_ChIP_HAM
195875096 208661072 3274163_G401_RBBP4ab79416_siCtrl_3_ChIP_HAM
286677171 302320690 3274164_G401_RBBP4ab79416_siPHIP_1_ChIP_HAM
136390669 148315164 3274165_G401_RBBP4ab79416_siPHIP_2_ChIP_HAM
220049433 232790842 3274166_G401_RBBP4ab79416_siPHIP_3_ChIP_HAM
309469926 331236490 3274167_G401_MTA2ab8106_siCtrl_1_ChIP_HAM
309668521 326415250 3274168_G401_MTA2ab8106_siCtrl_2_ChIP_HAM
392433914 412955926 3274169_G401_MTA2ab8106_siCtrl_3_ChIP_HAM
236345319 246764534 3274170_G401_MTA2ab8106_siPHIP_1_ChIP_HAM
242425544 255771042 3274171_G401_MTA2ab8106_siPHIP_2_ChIP_HAM
200072012 208240496 3274172_G401_MTA2ab8106_siPHIP_3_ChIP_HAM
136822524 140990774 3274173_G401_input_siCtrl_1_ChIP_HAM
133695061 138024506 3274174_G401_input_siCtrl_2_ChIP_HAM
127226094 131275744 3274175_G401_input_siCtrl_3_ChIP_HAM
142727773 147675190 3274176_G401_input_siPHIP_1_ChIP_HAM
133539453 138644722 3274177_G401_input_siPHIP_2_ChIP_HAM
207273105 217783712 3274178_G401_input_siPHIP_3_ChIP_HAM
198482035 249086528 2695628_G401_HAcst3724S_GFP_1_ChIP_HAM
184917778 217852842 2695629_G401_HAcst3724S_GFP_2_ChIP_HAM
285025459 302836238 2695630_G401_HAcst3724S_HA-PHIP_1_ChIP_HAM
108363226 119641824 2695631_G401_HAcst3724S_HA-PHIP_2_ChIP_HAM
35490589 36963842 2667506_G401_H3K4me3ab8580_siCtrl_1_ChIP_HAM
69969991 71588127 2667507_G401_H3K4me3ab8580_siCtrl_2_ChIP_HAM
68002766 70660793 2667508_G401_H3K4me3ab8580_siCtrl_3_ChIP_HAM
71524600 73826593 2667509_G401_H3K4me3ab8580_siPHIP_1_ChIP_HAM
58494355 60294212 2667510_G401_H3K4me3ab8580_siPHIP_2_ChIP_HAM
20409587 20958923 2667511_G401_H3K4me3ab8580_siPHIP_3_ChIP_HAM
42285306 43447830 2667512_G401_H3K27me3cst9733S_siCtrl_1_ChIP_HAM
77651940 80244925 2667513_G401_H3K27me3cst9733S_siCtrl_2_ChIP_HAM
61216811 64941946 2667514_G401_H3K27me3cst9733S_siCtrl_3_ChIP_HAM
32694551 33654905 2667515_G401_H3K27me3cst9733S_siPHIP_1_ChIP_HAM
69246423 70795552 2667516_G401_H3K27me3cst9733S_siPHIP_2_ChIP_HAM
68067152 69722043 2667517_G401_H3K27me3cst9733S_siPHIP_3_ChIP_HAM
141625970 144225152 3404887_G401_H3K27ac-ab4729_control_1_ChIP_HAM
154315580 156904476 3404888_G401_H3K27ac-ab4729_control_2_ChIP_HAM

157757009 160257760 3404889_G401_H3K27ac-ab4729_control_3_ChIP_HAM
157055512 159810540 3404890_G401_H3K27ac-ab4729_PHIP-OE_1_ChIP_HAM
163720055 166478374 3404891_G401_H3K27ac-ab4729_PHIP-OE_2_ChIP_HAM
148733492 151367118 3404892_G401_H3K27ac-ab4729_PHIP-OE_3_ChIP_HAM
133866861 135509482 3404893_G401_H3K27ac-ab4729_PHIP-KD_1_ChIP_HAM
164479238 166929966 3404894_G401_H3K27ac-ab4729_PHIP-KD_2_ChIP_HAM
146471057 148648404 3404895_G401_H3K27ac-ab4729_PHIP-KD_3_ChIP_HAM
165131827 167388666 3404896_G401_H3K27ac-ab4729_PHIP-rescue_1_ChIP_HAM
149593916 151854492 3404897_G401_H3K27ac-ab4729_PHIP-rescue_2_ChIP_HAM
132430828 134316936 3404898_G401_H3K27ac-ab4729_PHIP-rescue_3_ChIP_HAM
197168570 202135128 3404915_G401_input_control_1_ChIP_HAM
194443455 199889006 3404916_G401_input_control_2_ChIP_HAM
203138515 208806024 3404917_G401_input_control_3_ChIP_HAM
153931954 157792622 3404918_G401_input_PHIP-OE_1_ChIP_HAM
164200929 169117526 3404919_G401_input_PHIP-OE_2_ChIP_HAM
167775723 172203026 3404920_G401_input_PHIP-OE_3_ChIP_HAM
181069170 185143680 3404921_G401_input_PHIP-KD_1_ChIP_HAM
183970331 189063664 3404922_G401_input_PHIP-KD_2_ChIP_HAM
159182677 162559510 3404923_G401_input_PHIP-KD_3_ChIP_HAM
177896532 181712982 3404924_G401_input_PHIP-rescue_1_ChIP_HAM
169369182 173046542 3404925_G401_input_PHIP-rescue_2_ChIP_HAM
185039109 189048708 3404926_G401_input_PHIP-rescue_3_ChIP_HAM
179758145 192262906 3128867_G401_PHIPmarc_siCtrl_1_ChIP_HAM
167619941 176533406 3128868_G401_PHIPmarc_siCtrl_2_ChIP_HAM
146695191 151813476 3128869_G401_PHIPmarc_siCtrl_3_ChIP_HAM
245087857 259312094 3128873_G401_PHIPmarc_siPHIP_1_ChIP_HAM
227227156 240214496 3128874_G401_PHIPmarc_siPHIP_2_ChIP_HAM
195177623 209015402 3128875_G401_PHIPmarc_siPHIP_3_ChIP_HAM
159131405 160083136 3128876_G401_PHIPinput_siCtrl_1_ChIP_HAM
171258369 171939596 3128877_G401_PHIPinput_siPHIP_1_ChIP_HAM
175270996 226419316 3334381_G401_CHD4ab240640_GFP_1_ChIP_HAM
160856331 183152540 3334382_G401_CHD4ab240640_GFP_2_ChIP_HAM
147552321 187391232 3334383_G401_CHD4ab240640_GFP_3_ChIP_HAM
161789695 179667278 3334384_G401_CHD4ab240640_SMARCB1_1_ChIP_HAM
206891548 250522558 3334385_G401_CHD4ab240640_SMARCB1_2_ChIP_HAM
203188039 235939914 3334386_G401_CHD4ab240640_SMARCB1_3_ChIP_HAM
118101328 127762080 3334387_G401_input_GFP_1_ChIP_HAM
126587418 136582776 3334388_G401_input_GFP_2_ChIP_HAM
115685601 125816444 3334389_G401_input_GFP_3_ChIP_HAM
102747180 110427844 3334390_G401_input_SMARCB1_1_ChIP_HAM
74614068 80772396 3334391_G401_input_SMARCB1_2_ChIP_HAM
117693352 126170568 3334392_G401_input_SMARCB1_3_ChIP_HAM
We Sequence to a depth of ~100M some sample are single end and others are paired-end. Read lengths may vary from 50bp to 75bp

Antibodies

Drosophila antibodies (Active Motif, 61751)

Anti-DDB1 (Rabbit Monoclonal, Abcam, ab109027, Lot 1018022-6) (1:1000 WB, 1:50 ChIP)
https://www.citeab.com/antibodies/761059-ab109027-anti-ddb1-antibody-epr6089

Anti-H3K14ac (Rabbit Monoclonal, Abcam, ab52946, Lot 1029948-24, 1001631-4) (1:2000 WB, 7:1000 ChIP)
https://www.abcam.com/en-us/products/primary-antibodies/histone-h3-acetyl-k14-antibody-ep964y-chip-grade-ab52946

Anti-H3K27ac (Rabbit Polyclonal, Abcam, ab4729, Lot 1058174-1) (1: 2000 WB, 5:1000 ChIP)
https://www.abcam.com/en-us/products/primary-antibodies/histone-h3-acetyl-k27-antibody-chip-grade-ab4729

Anti-CHD4 (Rabbit Monoclonal, Abcam, ab240640, Lot 1043705-7, 11211685-1) (1:1000 WB, 1:100 IP, 8:1000 ChIP)
https://www.abcam.com/en-us/products/primary-antibodies/chd4-antibody-epr22953-38-chip-grade-ab240640

Anti-RBBP4 (Rabbit Monoclonal, Abcam, ab79416, Lot GR3418888-4) (1:1000 WB, 1:100 IP, 1:100 ChIP)
https://www.abcam.com/en-us/products/primary-antibodies/rbbp4-antibody-epr3411-chip-grade-ab79416

Anti-HDAC1 (Rabbit Polyclonal, Active Motif, 40967, Lot 24046163-1) (1:1000 WB, 5:1000 ChIP)
https://www.activemotif.com/catalog/details/40967/hdac1-antibody-pab

Anti-MTA2 (Rabbit Polyclonal, Abcam, ab8106, Lot 1058517-8) (1:1000 WB, 5:1000 ChIP)
https://www.abcam.com/en-us/products/primary-antibodies/mta2-pid-antibody-ab8106

Anti-HA (Rabbit Monoclonal, Cell Signaling Technology, 3724, Lot 11) (15:1000 ChIP)
https://www.cellsignal.com/products/primary-antibodies/ha-tag-c29f4-rabbit-monoclonal-antibody/3724?srsltid=AfmBOooo_OoULVwUHsqUpyAY2p4NLMc40GMBTn7IFCn5KRL96WMn8NSa

Anti-H3K4me3 (Rabbit Polyclonal, Abcam, ab8580, Lot GR3425198-1) (5:1000 ChIP)
https://www.abcam.com/en-us/products/primary-antibodies/histone-h3-tri-methyl-k4-antibody-chip-grade-ab8580

Anti-H3K27me3 (Rabbit Monoclonal, Cell Signaling Technology, 9733, Lot 19) (1:50 ChIP)

https://www.cellsignal.com/products/primary-antibodies/tri-methyl-histone-h3-lys27-c36b11-rabbit-monoclonal-antibody/9733?srsltid=AfmBOoobqW7shMUPIyN19B5YN17DETdFaijyCPtoUb6VKvV6Q_CR7iHy

Anti-H4ac (Rabbit Polyclonal, Millipore, 06-866, Lot 4049811) (15:1000 ChIP)
https://www.merckmillipore.com/HK/en/product/Anti-acetyl-Histone-H4-Antibody,MM_NF-06-866

Anti-PHIP (Rabbit, Ali Shilatifard, Northwestern University) (1:50 ChIP)
https://pmc.ncbi.nlm.nih.gov/articles/PMC8653789/

Peak calling parameters	Peaks were called using MACS2 v2.2.7.1 with -nomodel -q 0.05 flags (high confidence peaks). Low-confidence narrow peaks were also called using a more relaxed criteria (-q 0.5 flag).
Data quality	We have rigorous methods to evaluate data quality. Peaks were called using MACS2 v2.2.7.1 with -nomodel -q 0.05 flags (high confidence peaks). Low-confidence narrow peaks were also called using a more relaxed criteria (-q 0.5 flag). Reproducible peaks of biological replicates were called using a previously reported approach. Reproducible peaks were defined as loci those with overlapping high-confidence peaks in more than one replicate, or loci called as a high-confidence peak in one replicate and high- or low-confidence peaks in all other replicates.
Software	Trim-Galore (v0.4.4,) cutadapt, FastQC, bwa aln bwa samse (v0.7.12-r103963), samtools, STAR (2.7.1a65), biobambam2 (v2.0.8766). SPP (v1.1167) bedtools (v2.24.068) UCSC tools (v469) MACS2 v2.2.7.1, pybedtools (v0.8.168,72), edgeR, limma-voom, R, deeptools, HOMER (v4.9.1), RSEM, BETA, GSEApy (v1.1.2). We performed an input scaled spike in normalization using custom code that can be accessed through this link: https://github.com/jamyers2358/Malone_PHIP_SWISNF_Dependency and is referenced in the Code Availability statement.

Flow Cytometry

Plots

Confirm that:

☐ The axis labels state the marker and fluorochrome used (e.g. CD4-FITC).

☐ The axis scales are clearly visible. Include numbers along axes only for bottom left plot of group (a 'group' is an analysis of identical markers).

☐ All plots are contour plots with outliers or pseudocolor plots.

☐ A numerical value for number of cells or percentage (with statistics) is provided.

Methodology

Sample preparation	Tumor organoids were prepared in a 5 mL FACS tube in 10% BSA in PBS for analysis
Instrument	BD LSRFortessa Cell Analyzer – 4 lasers, 17 colors
Software	DiVa, FlowJo v5.4+
Cell population abundance	N/A
Gating strategy	Tumor organoid cells were gated for single cells sized at 55, subsequent single cells were treated with DAPI for live/dead percentages. Of live cells, mCherry was used to determine percentage of infected cells.

☐ Tick this box to confirm that a figure exemplifying the gating strategy is provided in the Supplementary Information.
